# Supplementary material for: The genome sequence of Hyocomium armoricum (Brid.) Wijk & Margad. (Hypnales: Hypnaceae)
Source: Wellcome Open Res. 2026 Jul 7;11:425. [Version 1] doi: 10.12688/wellcomeopenres.26967.1 (PMC13560601; doi:10.12688/wellcomeopenres.26967.1)
Supplement: Peer review history

# Reviewer report: Reviewer response for version 1

**DOI:** 10.21956/wellcomeopenres.29688.r162197

**Published:** 2026-08-10

**Authors:** Marco Todesco

**Affiliations:** 1 Michael Smith Laboratories, University of British Columbia, Vancouver, Canada

The data note reports the generation of a genome assembly for the pleurocarpous moss *Hyocomium armoricum *(flagellate feathermoss). The genome assembly is, characteristically for Darwin Tree of Life assemblies, of high quality, completeness, and contiguity, and will undoubtedly prove to be an important resource for comparative studies on mosses and plants more generally.

 A few minor comments:

 - The text and figures provide all the necessary technical information and analyses. However, a little more information in a couple of places would be useful for a reader who is not familiar with the species or with DToL standard pipeline for genome assemblies. It would be useful to specify in the background section the size of individuals of *H. armoricum*, which would make it clear why the low-input pipeline was used (including the generation of shorter-than-ideal HiFI reads), and why a separate individual was used for Hi-C. As mentioned by another reviewer, it would also be important to clarify the implications of using different individuals to generate the primary sequencing reads and scaffolding information - structural variation at sex chromosomes or elsewhere in the genome could generate locally inaccurate scaffolding.

 - The Hi-C heatmap has a grey cross in the middle. It would be helpful to explain, in the legend of the figure, what is causing that.

 - Information about DNA sharing to 10 Kbp is repeated in the "Nucleic acid extraction" and "PacBio HiFi library preparation and sequencing" sections.

 - A couple of sections seem to have generic DToL language: "Prepared libraries with a molarity greater than 2 nM were normalised to 2 nM, and 15 μL was used for making complexes.", "Depending on sample concentration and biotinylation percentage determined at the crosslinking stage, libraries were amplified with 10–16 PCR cycles." Were multiple HiFi and Hi-C libraries prepared?

Are sufficient details of methods and materials provided to allow replication by others?

Yes

Is the rationale for creating the dataset(s) clearly described?

Yes

Are the datasets clearly presented in a useable and accessible format?

Yes

Are the protocols appropriate and is the work technically sound?

Yes

Reviewer Expertise:

Plant genetics and genomics. Plant adaptation.

I confirm that I have read this submission and believe that I have an appropriate level of expertise to confirm that it is of an acceptable scientific standard.

# Reviewer report: Reviewer response for version 1

**DOI:** 10.21956/wellcomeopenres.29688.r162194

**Published:** 2026-08-07

**Authors:** Peng-Fei Ma

**Affiliations:** 1 Chinese Academy of Sciences, Kunming, China

In this study, the genome of a moss ( *Hyocomium armoricum*) was sequenced. A total of 333.6 Mb sequences were assembled and scaffolded into 11 chromosomal pseudomolecules. The quality of the genome assembly is high as evidenced by BUSCO evaluation and assembly QV scores, among others. As the first sequenced genome of its genus,  it would be a valuable genomic resource for future studies.

Are sufficient details of methods and materials provided to allow replication by others?

Yes

Is the rationale for creating the dataset(s) clearly described?

Yes

Are the datasets clearly presented in a useable and accessible format?

Yes

Are the protocols appropriate and is the work technically sound?

Yes

Reviewer Expertise:

Plant genomics and evolution

I confirm that I have read this submission and believe that I have an appropriate level of expertise to confirm that it is of an acceptable scientific standard.

# Reviewer report: Reviewer response for version 1

**DOI:** 10.21956/wellcomeopenres.29688.r162196

**Published:** 2026-07-28

**Authors:** Ruslan Kalendar

**Affiliations:** 1 University of Helsinki, Helsinki, Finland

This Data Note reports a chromosome-level haploid genome assembly for *Hyocomium armoricum*, a pleurocarpous moss of oceanic streamside habitats, generated as part of the Darwin Tree of Life project. The assembly (GCA_964235365.1, cbHyoArmo16.1) spanned 333.60 Mb, with 99.59% of the sequence placed on 11 chromosomal pseudomolecules, a contig N50 of 2.68 Mb, a scaffold N50 of 28.97 Mb, consensus quality QV 61.4, and *k*-mer completeness of 99.79%. Mitochondrial (104.43 kb) and plastid (123.84 kb) genomes have also been reported.

 This is a technically strong assembly and a genuinely useful addition to the sparse genomic resources available for pleurocarpous mosses. The contiguity and consensus accuracy metrics are excellent, the data are properly deposited under an INSDC BioProject, the software versions are tabulated, and the sequenced material is backed by a citable herbarium voucher at E with a resolvable specimen URL. The standard of vouchering should be routine and often is not.

**1. **Hi-C data were generated from a **different individual** (cbHyoArmo3 / EDTOL03435) than the one used for HiFi sequencing (cbHyoArmo16 / EDTOL03448). This is an accepted DToL practice for small organisms, and the two were collected on the same occasion, but it carries a specific risk in this species that goes unremarked: the manuscript itself states that *H. armoricum* is dioicous, and moss shoot tissue is a haploid gametophyte carrying either a U (female) or V (male) sex chromosome.

 If the two specimens are of opposite sexes, the Hi-C data cannot correctly scaffold the sex chromosome of the HiFi individual, and the sex chromosome pseudomolecule may be misassembled, fragmented, or absent. Bryophyte sex chromosomes are typically repeat-rich and structurally distinctive.

**2. **The Genome sequence report states that "the sequencing data provided approximately 380× coverage.” This does not follow from the reported figures under any of the three genome size estimates given:

 Assembly span (333.60 Mb)

 79,580 / 333.6=239×

 GenomeScope2 (325.5 Mb)

 79,580 / 325.5=245×

 Flow cytometry (410.00 Mb)

 79,580 / 410.0=194×

 The GenomeScope2 profile in Figure 2 reports kcov:190 with p:1, that is, a haploid model with a *k*-mer coverage of 190×. I suspect that 380× has been read off the second (2n repeat) peak visible at 380 in Figure 2 or carried over from a diploid model template. Please recalculate and state the genome size estimate on which the figure is based.

**3. **The text reports "44 breaks and 49 joins. This reduced the scaffold count by 49.9% and reduced the total assembly length by 1.7%."

 44 breaks and 49 joins resulted in a *net* change of only approximately five scaffolds. A 49.9% reduction implies that the scaffold count fell from approximately 593 to 297, that is, approximately 296 scaffolds disappeared, and a 1.7% length reduction implies that approximately 5.8 Mb of sequence was removed. Neither is explicable by breaking and joining alone; the sequence must have been deleted (contaminant scaffolds, haplotypic duplicates, or organelle-derived contigs).

**4. **The blob plot legend reports total [299; 334M], Streptophyta [13; 327M; 29M], and no-hit [286; 6.44M; 23.6k]. However, the 11 pseudomolecules in Table 3 sum to 332.25 Mb, which is against the 333.60 Mb span, leaving only 1.35 Mb outside the chromosomes, not 6.44 Mb. There is an apparent discrepancy of approximately 5 Mb between the two accountings.

 Please check and reconcile these numbers or explain the difference (for example, if BlobToolKit applies to a length or coverage filter or if the 13 Streptophyta-assigned scaffolds are not the 11 chromosomes plus the two organelles). Relatedly, 286 unplaced no-hit scaffolds is a substantial residue for an assembly of this quality; a brief characterisation would be welcome.

Are sufficient details of methods and materials provided to allow replication by others?

Yes

Is the rationale for creating the dataset(s) clearly described?

Yes

Are the datasets clearly presented in a useable and accessible format?

Yes

Are the protocols appropriate and is the work technically sound?

Yes

Reviewer Expertise:

comparative genomics, bioinformatics

I confirm that I have read this submission and believe that I have an appropriate level of expertise to confirm that it is of an acceptable scientific standard.
